# Temporal Trends in Mortality from Gunshot Wounds for Emergency Medical Services Compared to Private Vehicle Transport

**DOI:** 10.5811/westjem.53151

**Published:** 2026-06-28

**Authors:** Gregory Muller, Kate Miller, Loretta Matheson, Xinhang Wang, Laleh Gharahbaghian, Peter D’Souza, Marc Gautreau, Maame Yaa A. B. Yiadom

**Affiliations:** * Stanford University, Department of Emergency Medicine, Section of Prehospital and Disaster Medicine, Palo Alto, California; † Stanford University, Quantitative Sciences Unit, Palo Alto, California; ‡ Stanford University, Department of Emergency Medicine, Study Design and Statistical Analysis Core, Palo Alto, California; § Stanford University, Department of Emergency Medicine, Clinical Operations and Management, Palo Alto, California; || Emergency Care Health Services Research Data Coordinating Center, Department of Emergency Medicine, Palo Alto, California

## Abstract

**Introduction:**

Despite expectations that emergency medical services (EMS) transport following a gunshot wound (GSW) would improve 30-day mortality compared to transport by private vehicle, the opposite has been found in several studies. In response, EMS has adopted interventions over the past decade to reduce patient mortality. We compared mortality of GSW patients transported by EMS versus private vehicle as these field practice advancements became widespread.

**Methods:**

We conducted a retrospective cohort study using data from the National Trauma Data Bank (NTDB) from 2007–2021. We included adults (≥ 16 years of age) with GSW injuries transported solely by ground EMS (≤ 2 hours) or by personal vehicle to a Level I or Level II trauma center. The primary outcome was 30-day in-hospital mortality. We also aimed to describe demographic changes over time in these patient populations. A multivariable logistic regression model was developed to estimate the adjusted odds ratio (aOR) of mortality, with demographic and clinical controls. To describe change over time, we performed the multivariable logistic regression model separately within three-year periods. To determine whether the change was significant, we performed the multivariable logistic regression model with the same controls and the addition of an interaction term for transport mode with year group.

**Results:**

Of 355,913 patients, 83.7% were transported by EMS and 16.3% by private vehicle. Unadjusted mortality was consistently higher for EMS patients, ranging from 18.8% (vs 3.7% private vehicle) in 2007–2009 to 19.3% (vs 4.3% private vehicle) in 2019–2021. The aOR for mortality in the EMS cohort decreased from 2.2 (95% CI, 1.7–2.8) in 2007–2009 to 1.6 (95% CI, 1.3–1.8) in 2019–2021. However, this temporal trend was not statistically significant (P = .17).

**Conclusion:**

Our analysis suggests EMS transport remains associated with higher odds of mortality compared to private vehicle transport for patients with gunshot wounds. This may suggest that improvements in EMS clinical and transport practices have not yet proved sufficient to reduce relative mortality, or that there may be differences in injury severity or type of injury that are not adequately accountable from the NTDB data.

## INTRODUCTION

### Background

A landmark 2014 study showed that gunshot wound (GSW) patients transported by emergency medical services (EMS) to trauma centers had twice the odds of in-hospital mortality compared to patients transported by private vehicle.^1^ That study used data from the National Trauma Data Bank (NTDB), included four years of data from 2007–2010, and used the same control variables as our study except for the use of the New Injury Severity Score instead of the Injury Severity Score (ISS) and with the addition of the patient’s need for ventilatory support. Previous single-center studies evaluating mortality by transport mode showed mixed results.^2^,^3^

### Importance

The association of transport mode after GSW with survival is complex. It is likely that EMS transports a population with higher acuity and injury severity, as patients who are hemodynamically unstable or incapacitated are unable to transport themselves. Therefore, higher mortality in the EMS cohort may reflect this selection bias rather than a failure of prehospital care. It is also critical to acknowledge that GSW injuries do not occur in isolation but in communities, and they disproportionately impact racial and ethnic minorities. This reflects the underlying structural disparities that persist outside trauma systems of care. The transport decisions analyzed in this study must be understood within a context of unequal exposure to violence.

Advancements in understanding the complex associations at play have resulted in EMS protocols focused on shortening EMS on-scene and transport intervals to enable more of the early life-saving time window to occur at the destination hospital where there is additional access to life-saving interventions (ie, surgery). In parallel, EMS protocols have focused on bringing life-saving hospital interventions such as tourniquets and tranexamic acid (TXA)^4^–^8^ to the field as research identified the potential of these interventions to reduce mortality.^9^,^10^

### Goals of This Investigation

Our study evaluated 15 years of NTDB data from 2007 to 2021—extending the span of the seminal work an additional 11 years during which significant developments in EMS field practice have occurred. Our primary goal was to assess whether EMS mortality-reducing efforts were associated with reduction in the relative mortality of GSW patients transported by EMS compared to private vehicle. Other researchers^11^–^13^ using NTDB data to examine the relative mortality of patients transported by EMS versus private vehicle did not allow for direct comparisons due to variations in study design. Our study extends prior findings by performing a longitudinal analysis using consistent methods across the entire period from 2007–2021, a period marked by widespread adoption of hemorrhage control adjuncts and modernized trauma treatment protocols. Unlike replication studies, our objective was to determine whether these efforts coincided with a narrowing of the survival gap between EMS and private vehicle transport. We recognize that EMS transport may be disproportionately used for patients with higher clinical acuity; however, we hypothesized that advancements in prehospital care would sufficiently mitigate this difference to narrow the mortality gap between EMS and private vehicle transport over the study period. This would be consistent with a positive effect of changes to prehospital care over the time frame under study.

**Population Health Research Capsule**

What do we already know about this issue?

*Gunshot wound (GSW) patients transported by emergency medical services (EMS) have had twice the odds of mortality compared to private vehicle transport*.

What was the research question?

*Is there a persistent mortality gap between EMS and private vehicle transport for GSW trauma?*

What was the major finding of the study?

*Despite changes to EMS protocols over the last 15 years, a higher mortality persists for EMS transported gunshot wounds vs. private vehicle transports (aOR of mortality 1.6 in 2019–2021, CI 1.3–1.8)*.

How does this improve population health?

*Transport by EMS has a persistently higher mortality compared to private vehicle transport, highlighting an ongoing need for EMS system improvement*.

## METHODS

### Study Design

We developed a multivariable logistic regression model of the relationship between mortality and transport for GSW trauma patients from 2007–2021. We posited that if changes to EMS practices in the field and during transport have led to reduced mortality, then the odds of 30-day in-hospital mortality for patients transported by EMS compared to private vehicle should have improved over time. We also describe changes in the demographic and clinical characteristics of GSW trauma patients transported by EMS versus private vehicle over this full time frame. The protocol received institutional review board (IRB) exemption from the Stanford University IRB (#74250).

### Setting

We used data from the NTDB, a national database maintained by the American College of Surgeons (ACS). The NTDB is the largest U.S. trauma registry and includes data on patient demographics, injury classification, EMS prehospital intervals, clinical variables present on patient arrival, and outcomes. The Trauma Quality Program (TQP) Participant Use Files (PUF) are the representative data files provided by the ACS and contain all records sent to the NTDB for any specified year.

### Selection of Participants

We included adult patients (≥ 16 years of age), transported solely by either ground EMS or private vehicle, who initially presented to any Level I or Level II hospital (regardless of volume) with GSW injuries only. We analyzed the TQP PUF data files for admission years 2007–2021. Note that 2020 was the last year for which EMS transport interval data was collected. We excluded patients who were transferred from another hospital or transferred out to another hospital within 30 days. We also excluded patients transported by police. When EMS transport intervals were available, we established a cut-off duration of two hours and excluded patients with transport times above that threshold as outliers.

Patients with GSWs were identified using the TQP’s external cause of injury codes (e-codes, based on *International Classification of Diseases* codes) that TQP further categorizes into mechanism of injury. We included only those patients with a “Mechanism of Injury = Firearm,” and only if any secondary mechanism of injury was not a different type of injury, such as a fall.

### Outcomes

The primary outcome was death within 30 days during the hospital admission, or survival to hospital discharge. To determine whether the patient either died in hospital or was discharged alive within 30 days, we used the following variables: ED discharge disposition; hospital discharge disposition; ED length of stay (LOS) (2017–2021 only); and hospital LOS. If ≥ 30 days, we used as a proxy for hospital LOS the following: ICU LOS; days in the hospital on a ventilator; and days to withdrawal of life support. For 2007–2010, the variable EDDEATH was used as a secondary determination of death in the ED.

### Measurements

We describe the population clinical and demographic characteristics with means, medians, and percentages as appropriate, separated by transport type (EMS versus private vehicle). We also evaluated these characteristics within sequential time periods to examine any compositional changes to the population over time. We did not test for statistically significant differences in characteristics between EMS and private vehicle patients to reduce the likelihood of a type I error that would result from running many statistical tests. Neither did we test for these differences because the large sample size (≥ 300,000) has statistical power to detect very small differences between groups, which may achieve statistical significance but carry little to no clinical or substantive meaning.

### Data Analysis

We estimated the adjusted odds ratio (aOR) of 30-day mortality with multivariable logistic regression models. The predictor of interest was the binary variable for transport: EMS versus private vehicle. Control variables included patient age, sex, race and ethnicity, insurance status, ISS,^14^,^15^ and on presentation to the trauma center, the initial systolic blood pressure (SBP), heart rate, and motor component of the Glasgow Coma Scale (GCS). To test for statistically significant change in aOR over time, we report the *P* value of an interaction term between time period and transport type using all time periods pooled in one dataset. To facilitate interpretation of changes in aOR over time, a separate model was fit for each of five time periods: 2007–2009; 2010–2012; 2013–2015; 2016–2018; and 2019–2021.

Our primary analysis was complete case, reflecting that the missingness on any covariate was 3.2% at most (see Table 1). As a post-hoc sensitivity analysis, our study entailed multiple imputation to impute values on the six predictors with any missingness: gender; race; ISS; BP; heart rate; and GCS). We ran the primary model on each of five replicates and combined the estimated parameters using the Rubin rules. *P* < .05 was considered to be significant for all statistical tests. All analyses were conducted with SAS Enterprise Guide v8.1, update 2 (SAS Institute Inc, Cary, NC). The statistical analysis plan registered at Open Science Foundation prespecified all analyses (https://osf.io/ah4fb/). This manuscript was prepared according to the Strengthening the Reporting of Observational Studies in Epidemiology (STROBE) reporting guidelines.

## RESULTS

**Figure 1:**
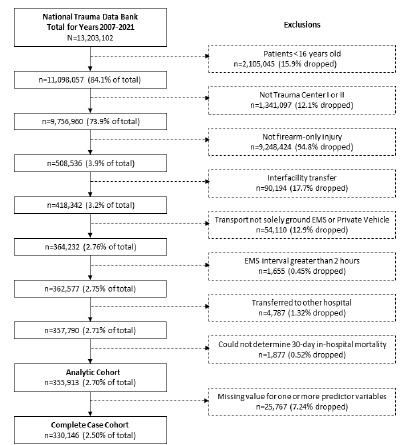
Consort diagram of study population from the National Trauma Data Bank in a study comparing mortality of gunshot wound victims transported by emergency medical services versus private vehicle. *EMS*, emergency medical services.

Of 13,203,102 records in the NTDB for the years 2007–2021, the sample included 355,913 patients (2.7%) who met the inclusion criteria (Figure 1 and Supplement Table S1) and 330,146 patients who were not missing data among the model covariates used in the regression model. Of these, 297,733 (83.7%) were transported by EMS and 58,180 (16.3%) by private vehicle. Table 1 shows the demographic and clinical characteristics of the patient population by transport type. The unadjusted mortality for patients in the EMS group was 19.1% (*n* = 56,798), and 4.0% (*n* = 2,321) for the private vehicle group. The EMS patients were more likely to be older, a race/ethnicity other than Black, and female compared to private vehicle patients. On arrival at the trauma center, the injuries of EMS patients were clinically more severe, as measured by ISS, and EMS patients were in more critical condition, as measured by GCS-motor.

**Figure 2:**
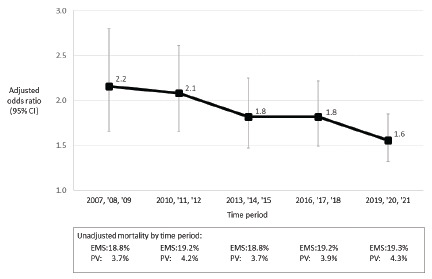
Adjusted and unadjusted 30-day in-hospital mortality for patients transported by emergency medical service relative to private vehicle by study period; odds ratios adjusted for demographic and clinical characteristics in a study of 15 years of National Trauma Data Bank data on mortality outcomes after gunshot wounds. *EMS*, emergency medical services; *PV*, private vehicle.

The complete case multivariable model, which controlled for demographic and clinical characteristics and included the full-time span of 2007–2021, found an overall higher mortality rate for patients transported by EMS compared to private vehicle. To facilitate interpretation, we present further results using separate models for the five time periods. Figure 2 presents the aOR and 95% confidence intervals of mortality for patients transported by EMS compared to private vehicle for each time period, adjusted for demographic and clinical characteristics. Although the estimated aOR fell from 2.2 in 2007–2009 (95% CI, 1.7–2.8) to 1.6 in 2019–2021 (95% CI, 1.3–1.8), this decline was not statistically significant *(P =* .17 for the interaction term of time and transport type in a model using all periods pooled). In the post-hoc sensitivity analysis using multiply imputed data, the interaction between time and transport type was highly significant at *P* = .004. Further diagnostics revealed that for certain covariates, the missingness was not at random but associated with both the exposure and outcome (Supplemental Table S2).

**Figure 3:**
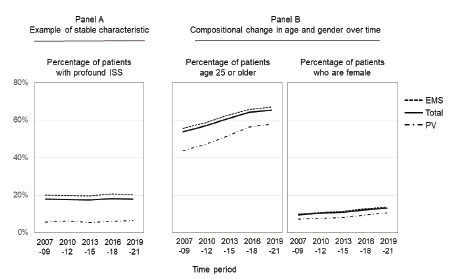
Selected characteristics of the gunshot wound trauma population by study period in our analysis across 15 years of National Trauma Data Bank data on mortality outcomes after gunshot wounds, *EMS*, emergency medical services; *ISS*, Injury Severity Score; *PV*, private vehicle.

Many characteristics of the study population were stable across the study period for both EMS- and private vehicle-transported patients, including missing data patterns (Supplemental Table S3). This supplemental table also offers an understanding of univariate associations of mortality of EMS compared to private vehicle. The proportion of patients with profound ISS varied by less than one percentage point for both EMS patients (range 20.1%–20.3%) and private vehicle patients (range 5.6%–6.4%) (Figure 3). Two characteristics changed similarly for both EMS- and private vehicle-transported patients over time: The population became older, with the percentage of patients > 24 years of age rising from 55.6% to 66.8% among EMS transports and 43.7% to 57.8% among private vehicle transports, and the female percentage rose, from 9.9% to 13.6% among EMS transports and 7.1% to 10.4% among private vehicle transports (Figure 3).

## DISCUSSION

We found that between 2007–2021, the aOR of mortality among GSW trauma patients was significantly higher among patients transported by EMS than those transported by private vehicle, adjusting for demographic and clinical characteristics. The aOR point estimates of mortality for EMS-versus private vehicle-transported patients did decline over time, although the decline was not statistically significant in complete case analysis, which does not support our initial hypothesis. The analysis using multiply imputed data did show a statistically significant decline. However, we observed patterns suggesting the missingness was not at random, which introduces systematic bias into the regression estimates, making it more likely to find a significant difference that is an artifact of the imputation. In Supplement Table S2, we characterize the bias to enhance awareness of this in the literature. Our imputed analysis was a post-hoc sensitivity analysis. For these reasons, we placed less weight on its findings, found the complete case analysis to be more reliable, and considered the decline to not be statistically significant.

The finding of higher aOR of mortality among patients transported by EMS is consistent with other research,^1^,^11^–^13^ but our study is the first to measure the aOR of mortality longitudinally over an expanded time frame. In previous work focused on urban trauma systems, GSW patients transported by EMS were roughly twice as likely to die as those transported by private vehicle.^11^ Studies looking at expanded modes of transport again found roughly double the adjusted odds of mortality for EMS transport versus police or private vehicle.^12^ Finally, a study focused on torso-penetrating wounds found that the adjusted odds of mortality for EMS-transported patients was only 1.6 times that of private vehicle transport.^13^

We explored whether these results could have arisen from compositional changes in the population of GSW trauma patients over time. We found similar demographics to those reported by longitudinal studies conducted nationally^16^,^17^ and at individual Level I trauma centers^18^–^20^ throughout the United States covering the past two decades. Our study identified a shift toward an older (≥ 25 years of age) and increasingly female population, while other characteristics remained stable. The consistently high proportion of certain racial-ethnic groups in this population points to persistent disparities in GSW trauma exposure that warrants further investigation.

An important consideration in our results is the observed difference in patient populations between those transported by EMS versus private vehicle. Emergency medical services transported nearly five times as many patients as private vehicle, and these patients were more severely injured, with higher ISS, lower GCS–motor scores, and greater likelihood of low blood pressure and high heart rate on presentation to the trauma center. Given these differences, higher unadjusted mortality for the EMS transport cohort is to be expected.

### Prehospital Interventions and Mortality

Prehospital interventions with the aim of decreasing mortality include the use of tourniquets, which have been found to be life-saving among military personnel^4^,^5^ and effective at preventing exsanguination among civilians.^7^ In a study of civilian public mass shootings, injuries to the extremities were found to be the most common and the most frequently associated with vascular injury.^21^ Similarly, CRASH-2,^10^ a landmark study in trauma care, demonstrated the effectiveness of TXA in preventing all-cause mortality; further research demonstrated the effectiveness of prehospital TXA at reducing early mortality after trauma.^8^ In urban contexts, certain prehospital procedures, including intubation, needle decompression, tourniquet placement, cricothyroidotomy, and Advanced Cardiovascular Life Support care, were associated with a lower incidence of mortality in severely injured trauma patients and did not delay transport to definitive care.^9^ Blood administration was associated with a significant reduction in the odds of long-term mortality among all trauma patients but was inconclusive about hemorrhagic trauma patients.^22^

However, other prehospital procedures—or the extra time required to perform them—are associated with higher mortality. Seamon et al^23^ found that performance of prehospital procedures was the sole independent predictor of mortality: For each prehospital procedure performed, the patient was 2.6 times more likely to die before hospital discharge. Other studies demonstrated that select interventions thought to be beneficial, such as. endotracheal intubation^24^ and intravenous crystalloid administration,^25^,^26^ were either not associated with a mortality benefit or were detrimental. A 2018 meta-analysis found that longboard spinal immobilization was associated with increased mortality in penetrating trauma and had no benefit in mitigating neurologic defects.^27^

### Scene and Transport Intervals and Mortality

Shorter transit intervals may be a key driver of the lower mortality rates observed for private vehicle transports; however, transit intervals in the NTDB are not available for private vehicle cases and are largely missing for EMS. Previous work on this question using NTDB data has emphasized this limitation^1^,^11^–^13^,^28^

Longer transit time has been considered a cause of increased mortality, and previous work has found positive associations between mortality and transit times for penetrating injury trauma patients.^29^ This association has been demonstrated for general trauma,^30^ penetrating injuries,^31^ specifically thoracic,^32^ and cardiac^33^ penetrating injuries. However, other findings differ. A single-center study found that after an intervention that reduced transport times by seven minutes on average for abdominal vascular injuries, mortality increased dramatically.^34^ Longer scene times were associated with lower mortality for severe trauma patients^35^ or not associated with increased 30-day mortality after adjustment for other variables.^36^

### Study Results in Context

Since the initial 2010 finding that the mortality rates were higher for GSW patients transported by EMS compared to those transported by private vehicle,^1^ efforts have been made to eliminate time-wasting EMS interventions^27^,^37^ and to increase only those EMS practices associated with a mortality benefit.^7^,^8^,^10^,^23^,^38^ However, our study found only a slight, but non-significant, improvement in the aOR for mortality of GSW patients transported by EMS compared to those transported by private vehicle.

Unlike prior NTDB analyses that provided brief snapshots of trauma outcomes, this longitudinal study captures a period spanning significant evolution of prehospital trauma care. Despite the proliferation of advanced prehospital interventions (eg, TXA, tourniquets), the survival advantage associated with private vehicle transport has persisted. For this analysis and others using NTDB data,^1^,^11^,^13^,^28^ exploration of transit time has been hampered by the high and varying rates of missing data. We attempted to investigate the EMS transport and scene intervals, but the rate of missingness—higher than 50% in some years—prevented making reliable assertions about changes in EMS intervals over time.

## LIMITATIONS

Assuming the total duration of the prehospital interval (ie, time of injury to arrival at ED) is a central factor in the association with mortality, the absence of timing for this interval among patients transported by private vehicle presented a challenge. One study did establish the interval time between injury and arrival at a single large urban trauma center for both EMS-transported and private vehicle-transported patients and found surprisingly little difference in transport intervals, except for more critically injured patients (ISS ≥ 13) whose transport by EMS was slower than for those transported by private vehicle.^3^

The NTDB data also limited our analysis in several ways. First, the data do not capture patients who were first transported to a non-trauma hospital due to proximity, ignorance, clinical severity, or error, and who died at that location. Second, not all trauma centers contribute data to the NTDB, and each center is responsible for its data quality, which may have affected the availability of complete cases relied upon for this analysis. Third, we were unable to adjust for clustering at the level of the transporting agency or hospital because the NTDB does not provide unique EMS agency identifiers, and we were not able to obtain hospital identifiers. Clustering would not affect the point estimates but would likely widen the confidence intervals, which would not change our conclusion of no significant decline.

Fourth, several potential confounders are not captured in the NTDB: prehospital interventions; hospital-level quality of care; delays in EMS activation; distance to hospital; total estimated blood loss volume; complete details of transfusion requirements or surgical procedures; the caliber, number, and energy of projectiles inflicting injury; and the setting in which such trauma occurred. Last, in the course of our analysis we discovered that several clinical and demographic variables suggest missingness not at random with respect to transport type and 30-day mortality.

Excluding data from Level III and IV trauma centers from the study limits the generalizability of our results. We used the ISS to control for severity of injury, which is a widely used approach. However, there may be other aspects of injury severity that are not captured by this rating method. For example, the ISS categorizes a “severe” injury to the head, neck, chest, or abdomen equally to a “severe” injury to an extremity. However, the clinical challenges of hemorrhage control and hemorrhagic shock differ significantly across these body regions, with substantially different mortality outcomes. The ISS does not apply weighted adjustments to account for these differences, thus limiting its effectiveness as a control for clinically significant injuries with a higher likelihood of mortality. The observational design of our study cannot establish a causal relationship between the mode of transport and mortality outcome.

Efforts to reduce the excess mortality among GSW patients associated with EMS transport must continue. A primary improvement would be the deterministic linkage of prehospital registries (eg, National Emergency Medical Services Information System) with hospital outcomes data (eg, NTDB). Such longitudinal datasets would allow for the analysis of specific prehospital interventions, such as the exact timing of tourniquet application or duration of the prehospital interval, and their impact on hospital outcomes such as mortality. Crucially, this linkage would enable multilevel analyses that cluster by EMS agency to determine whether prehospital practice heterogeneity (eg, staffing models, agency type, protocol variability) are associated with observed mortality differences.

Additional research identifying and quantifying the effectiveness of existing or novel prehospital interventions (eg, prehospital blood administration) and their impact on mortality can help advance the growing knowledge of what improves survival. Improving the robustness of injury severity ratings so that they are validated for alignment with medical intervention intensity and mortality risk in situations of active hemorrhage can improve our ability to understand survival epidemiology. Causal research into this question is not possible because data cannot be collected on prehospital care of personal vehicle-transported patients, and randomization is obviously impossible. However, improving the quality and scope of observational data would support stronger observational designs.

Educational research on best methods to effectively train EMS clinicians in caring for trauma patients could also help improve patient mortality by better matching field practice with optimal interventions. Finally, additional research is needed on the structural disparities driving transport decision, specifically examining how race, socioeconomic status, and community violence influence the likelihood of EMS versus personal vehicle transport.

## CONCLUSION

In this retrospective analysis of gunshot wound patients, EMS transport remained associated with higher odds of mortality compared to private vehicle transport. Despite reported improvements to EMS clinical and transport practices over the 15-year study period, we did not observe a statistically significant narrowing of the relative mortality gap between transport modes, highlighting an ongoing opportunity for EMS system improvement. These findings represent associations and not causal relationships. Deterministic linkages between prehospital and in-hospital trauma registries represents a valuable improvement in determining the effectiveness of prehospital interventions and their impact on mortality. There is little evidence of a shift in the demographic composition of the GSW population except for a small increase in older patients (≥ 25 years of age) and female patients.

## Supplementary Information



## Figures and Tables

**Table 1:** Demographic and clinical characteristics of patients by transport type.

	EMS transport		PV transport	
	
	*n*	%	*n*	%
Total	297,733	83.7	58,180	16.3
Patient died in hospital ≤ 30 days				
No	240,935	80.9	55,859	96.0
Yes	56,798	19.1	2,321	4.0
Age, in years				
16–19	45,371	15.2	10,872	18.7
20–24	65,478	22.0	16,593	28.5
25–29	52,580	17.7	12,163	20.9
30–39	66,071	22.2	11,781	20.3
40–49	34,710	11.7	4,151	7.1
50–59	19,911	6.7	1,720	3.0
60–89	13,612	4.6	900	1.5
Age, median [IQR], in years	28 [22–38]		25 [21–32]	
Sex				
Female	35,709	12.0	5,180	8.9
Male	261,540	87.8	52,862	90.9
Race and Ethnicity				
Black	165,989	55.8	39,562	68.0
White	69,553	23.4	7,736	13.3
Hispanic/Latino	43,109	14.5	7,674	13.2
Asian	2,622	0.9	391	0.7
American Indian or Alaska Native	1,181	0.4	194	0.3
Other, multiple races, NHOPI*	9,138	3.1	1,602	2.8
Insurance status				
Private Insurance or Medicare	67,413	22.6	12,154	20.9
Medicaid or other government	92,794	31.2	19,154	32.9
Uninsured (Self Pay)	100,942	33.9	20,656	35.5
Other or Unknown	36,584	12.3	6,216	10.7
Injury Severity Score				
Mild: 1–8	109,143	36.7	34,596	59.5
Moderate: 9–15	88,983	29.9	15,750	27.1
Severe: 16–24	38,532	12.9	4,224	7.3
Profound: 25–75	59,889	20.1	3,513	6.0
Systolic blood pressure				
Normal	146,637	49.3	29,261	50.3
High: > 140	94,387	31.7	23,693	40.7
Low: < 90	19,718	6.6	2,852	4.9
Zero	26,879	9.0	1,176	2.0
Heart rate				
Normal	152,928	51.4	29,341	50.4
High: > 100	100,503	33.8	24,968	42.9
Low: < 60	10,014	3.4	1,726	3.0
Zero	26,528	8.9	1,138	2.0
Glasgow Coma Scale - Motor				
Appropriate response	225,560	75.8	53,459	91.9
Localizing pain	6,524	2.2	574	1.0
Withdrawal from pain	3,795	1.3	272	0.5
Flexion to pain	1,199	0.4	63	0.1
Extension to pain	1,227	0.4	67	0.1
No motor response	53,419	17.9	2,235	3.8
Year groupings				
2007, 2008, 2009	41,477	13.9	7,569	13.0
2010, 2011, 2012	52,219	17.5	9,216	15.8
2013, 2014, 2015	54,936	18.5	10,087	17.3
2016, 2017, 2018	64,923	21.8	12,987	22.3
2019, 2020, 2021	84,178	28.3	18,321	31.5

* NHOP = Native Hawaiian or other Pacific Islander.

Missing data as follows: sex, 0.2%; race and ethnicity, 2.0%; Injury Severity Score, 0.4%; systolic blood pressure, 3.2%, heart rate, 2.5%; and Glasgow Coma Scale–motor, 2.1%.

*EMS*, emergency medical services; *PV*, private vehicle.
