# Supplementary material for: Temporal Trends in Mortality from Gunshot Wounds for Emergency Medical Services Compared to Private Vehicle Transport
Source: West J Emerg Med. 2026 Jun 28;27(4):938–46. doi: 10.5811/westjem.53151 (PMC13436646; doi:10.5811/westjem.53151)
Supplement: Supplementary file 1 [file wjem-27-938-s001.pdf]

SUPPLEMENTAL TABLES

Table S1 – Details of study population consort diagram overall and by year groups

Table S2 – Evidence of Missing Not at Random (MNAR)

		Level of Missingness		% Arrived by EMS[2]	% Died in 30 days[2]
		N	As % of analytic cohort[1]		
Complete Cases				83.7%	16.6%
Cases missing on demographic variables	Gender	604	0.2%	77.6%	17.9%
	Race	7,162	2.0%	85.7%	32.9%
Cases missing on clinical variables	ISS	1,283	0.4%	92.4%	45.1%
	BP	11,310	3.2%	89.4%	60.8%
	Pulse	8,767	2.5%	88.5%	56.6%
	GCS	7,519	2.1%	79.9%	27.1%

[1] Analytic cohort N = 355,913

[2] Every encounter in the analytic cohort has data for these variables.

Table S3 - Demographic and clinical characteristics of patients by time period and transport type

		'08, '09			'11, '12			'14, '15			'17, '18			'20, '21		
	MS versus % PV		84.4	15.6		84.8	15.2		84.3	15.7		82.8	17.2		82.1	17.9
		% of category, by column														
ys	No	83.6	81.3	96.3	83.1	80.8	95.8	83.6	81.2	96.3	83.4	80.8	96.1	83.4	80.7	95.8
	Yes	16.4	18.8	3.7	16.9	19.2	4.2	16.4	18.8	3.7	16.7	19.2	3.9	16.6	19.3	4.3
ears	16-19	21.0	20.2	25.2	17.7	17.1	20.9	14.2	13.6	17.0	14.1	13.6	16.4	14.6	13.9	17.4
	20-24	25.2	24.2	31.1	25.5	24.3	32.0	25.1	23.9	31.6	21.8	20.7	27.4	20.2	19.2	24.9
	25-29	17.2	17.1	18.1	17.3	17.1	18.5	18.0	17.6	20.1	19.1	18.3	22.9	18.6	17.8	22.3
	30-39	18.7	19.1	16.6	19.8	20.0	18.6	21.2	21.5	19.7	22.6	23.0	20.8	24.5	24.9	22.5
	40-49	10.1	10.9	5.7	10.3	11.0	6.1	10.8	11.5	6.8	11.1	11.8	7.4	11.7	12.4	8.3
	50-59	4.9	5.4	2.2	5.9	6.5	2.7	6.5	7.1	3.2	6.5	7.1	3.1	6.1	6.8	3.1
	60-89	2.8	3.1	1.1	3.6	4.0	1.3	4.2	4.7	1.5	4.9	5.4	2.0	4.3	4.9	1.6
	Female	9.5	9.9	7.1	10.3	10.8	7.5	10.9	11.4	8.1	12.2	12.7	9.4	13.0	13.6	10.4
	Male	90.5	90.1	92.9	89.7	89.2	92.5	89.0	88.5	91.8	87.8	87.3	90.6	86.5	85.9	88.9
city	or African Amer	57.1	55.7	64.8	58.1	56.4	67.3	57.8	55.7	69.2	56.7	54.5	67.8	58.7	56.4	69.2

	White	18.4	19.6	11.7	21.5	22.9	13.4	23.4	25.1	14.5	23.9	25.6	15.4	20.7	22.6	11.8
	Hispanic/Latino	16.9	17.1	15.8	14.5	14.7	13.4	12.9	13.3	10.9	13.7	13.9	12.4	14.2	14.3	13.8
	Asian	1.1	1.1	1.3	0.9	1.0	0.8	0.8	0.9	0.7	0.8	0.9	0.6	0.7	0.8	0.4
	nd or Alaska Nat	0.3	0.3	0.2	0.3	0.3	0.3	0.3	0.3	0.3	0.4	0.4	0.3	0.5	0.5	0.5
	nultiple, NHOPI*	3.6	3.6	3.7	3.1	3.1	3.3	2.7	2.7	2.7	2.8	2.9	2.3	3.1	3.2	2.5
s	Ins or Medicare	16.2	16.5	14.2	19.8	20.0	18.6	23.2	23.5	21.4	25.5	25.9	23.7	23.9	24.2	22.5
	aid or Other Govt	20.3	20.4	19.4	25.4	25.6	24.6	28.6	28.4	29.5	35.2	34.9	36.7	39.4	38.8	41.9
	nsured (Self Pay)	38.5	37.7	42.5	37.4	36.7	41.5	35.5	35.0	38.1	32.4	32.2	33.4	30.7	30.9	29.7
	ther or Unknown	25.1	25.3	23.9	17.4	17.8	15.2	12.7	13.0	11.0	6.8	6.9	6.2	6.1	6.1	5.9
Score	Mild: 1-8	41.1	37.4	61.5	41.3	37.8	61.2	40.6	37.0	60.3	40.3	36.4	60.2	39.4	35.6	56.8
	Moderate: 9-15	28.1	28.7	24.8	28.3	28.8	25.6	29.8	30.3	27.0	29.4	30.0	26.4	30.5	30.8	29.3
	Severe: 16-24	12.1	13.0	7.6	12.1	13.0	6.8	11.9	12.8	7.0	11.8	12.7	7.3	12.2	13.2	7.5
	Profound: 25-75	17.9	20.1	5.6	17.7	19.7	6.2	17.4	19.6	5.5	18.2	20.7	6.0	17.8	20.3	6.4
	Normal	47.4	47.0	49.2	48.0	47.7	49.2	49.1	48.9	49.9	50.1	50.1	50.1	51.0	50.9	51.7
	High: > 140	35.1	34.1	40.5	35.1	33.8	42.2	34.4	32.9	42.1	32.3	30.6	41.0	31.0	29.3	39.1
	Low: < 90	6.0	6.1	5.0	5.9	6.0	4.9	5.8	6.0	4.5	6.5	6.8	4.7	7.1	7.5	5.2
	Zero	8.4	9.6	2.1	8.5	9.6	2.1	7.8	8.9	1.9	7.6	8.7	2.0	7.5	8.8	2.0
	Normal	52.0	52.5	49.7	52.3	52.5	50.8	51.8	51.9	51.3	50.8	50.9	50.3	50.2	50.2	50.1
	High: > 100	34.2	32.7	42.5	34.4	32.9	42.9	34.9	33.5	42.8	35.4	34.0	42.6	36.4	34.8	43.4
	Low: < 60	3.2	3.2	3.0	3.3	3.3	2.9	3.4	3.5	2.9	3.4	3.4	3.2	3.3	3.4	2.9
	Zero	7.9	9.0	1.9	8.1	9.2	2.1	7.6	8.6	1.8	7.6	8.7	2.0	7.7	9.0	2.0
Scale,	opriate response	78.2	75.9	90.3	78.5	76.2	91.9	78.6	76.0	92.9	78.2	75.4	92.1	78.5	75.6	91.8
	Localizing pain	1.7	1.9	0.7	1.9	2.0	1.1	2.1	2.3	0.8	2.0	2.2	1.0	2.2	2.4	1.2
	drawal from pain	1.1	1.2	0.5	1.1	1.2	0.5	1.2	1.3	0.4	1.2	1.3	0.4	1.2	1.3	0.5
	Flexion to pain	0.3	0.4	0.1	0.4	0.5	0.1	0.3	0.4	0.1	0.4	0.4	0.1	0.4	0.4	0.1
	Extension to pain	0.3	0.4	0.1	0.4	0.4	0.2	0.4	0.4	0.1	0.4	0.4	0.1	0.3	0.4	0.1
	motor response	15.3	17.4	3.7	15.9	17.9	4.1	15.4	17.5	3.6	15.9	18.3	3.9	15.7	18.2	3.9

Caption: NHOPI = Native Hawaiian or Other Pacific Islander
